# Digital twins and systemic acceleration: insights from Luhmann’s sociological perspective

**DOI:** 10.3389/fsoc.2026.1884848

**Published:** 2026-09-04

**Authors:** Simon Plakolb, Raffael Hiden

**Affiliations:** 1 Centre for Ecological and Evolutionary Synthesis, Institute of Biosciences, Faculty of Mathematics and Natural Sciences, University of Oslo, Oslo, Norway; 2 Department of Sociology, Faculty of Social and Economic Sciences, University of Graz, Graz, Austria

**Keywords:** acceleration, complex systems, complexity, digital twin, Luhmann, social systems, system

## Abstract

The digital age has brought about many hypes about new technology of which digital twins are among the most recent. Techno-optimistic thought proclaims them to bring about a revolution in societal management. We interrogate Luhmann’s sociology about the relationship between a social system and (its) digital twin(−s). In doing so we discover that common definitions of digital twins do not go beyond long-established technologies. If a digital twin is a computer model that is fed by information of the physical system which it influences in some feedback loop, to Luhmann every computer model is one. Yet, the term points to the apparent necessity to baptize an observable difference. We identify the latest and ongoing acceleration of integrated digital systems as this difference that makes a difference. This systemic acceleration, the maximization of the velocity of self-reference, builds the focal point in our discussion of looming changes. We think that digital twins encode a power relationship. Only if society remains in control of the modes of selection can a digital twin foster its second-order observation. To this end, the engineering derived notion of a direct cybernetic wire that connects the digital twin to the physical must be replaced with a mechanism for structural coupling. A social digital twin can then act as a digital mirror that enables an immediate, radicalized second-order observation. Thus, digital twins are in our understanding the epitome of the accelerative effect that digitalization has on society.

## 1 Introduction

Digital twins (DTs) are commonly defined in relation to a physical twin (PT) to which they are connected (Grieves, 2015; National Academies of Sciences, Engineering, and Medicine, 2024). Sensor information flows from the physical to the digital twin which in return controls the physical. They originated in engineering where DTs were first introduced by NASA to conduct virtual experiments but have since made their way to other fields (Grieves and Vickers, 2017). The idea and motivation behind them are both simple: Instead of trying policies in an engineered physical system, a digital replica can reveal policy consequences by virtue of simulation. Not only does this mitigate expensive physical damages but it also reduces the cost of policy inquiries rendering large scale, albeit virtual, experiments feasible (Grieves and Vickers, 2017).

Engineered systems, and especially those that were subject to the first adoption of DTs such as, for example, robot arms, are typically well understood and situated in a sterile environment. Their digital replica can be built using readily available blueprints. It is in this light, that we can understand early enthusiasm about a one-to-one correspondence between a digital twin and its physical twin. The first academic reports on DTs fall victim to the same techno-optimism. They propose the *mitigation of unpredictable behavior* and *models at the atom level* (Grieves and Vickers, 2017), or the aim to make DTs *completely indistinguishable* from their PTs (Rasheed et al., 2020). In the context of social systems these notions are almost absurd, and reminiscent of postmodernist theories of hyperreality and can be compared to the map of Borges’ “Del rigor en la ciencia” that literally covers its territory. However, in an age of high-resolution satellite imagery at everyone’s hands, it may appear realistic to ask for the impossible. In other words, the digital transformation promises to solve long standing paradoxa. And, as Luhmann said, the paradox is the orthodox of our times (Luhmann, 2021b, pp. 1144).

As a working definition for digital twins we use that of the National Academies of Sciences, Engineering, and Medicine (2024): “A digital twin is a set of virtual information constructs that mimics the structure, context, and behavior of a natural, engineered, or social system (or system-of-systems), is dynamically updated with data from its physical twin, has a predictive capability, and informs decisions that realize value. The bidirectional interaction between the virtual and the physical is central to the digital twin.” This definition is more compatible with DT applications on social systems than earlier ones, as it speaks of *mimic*ry rather than *models on an atom level*. Its final sentence emphasizes a *bidirectional interaction* of the physical and the digital twin which is not only central to the definition but will also show to be a vital factor in our analysis. In the scope of engineering this interaction is often envisioned as a direct control loop. As we will argue in the following, a sociological appropriation of DTs must instead reduce them to a mechanism for structural coupling.

Social systems theory proves to be the ideal link for this approach because it already includes the central theoretical elements. The challenge lies in applying these theoretical building blocks to current areas of digital society. Luhmann’s theory adapts the idea of autopoiesis, originally developed for biological systems, to the social sphere. Autopoiesis describes the constant reproduction of the self as an inside-out process of the system (Maturana and Varela, 1980). The system stabilizes its structure to construct a boundary that differentiates and links system and environment (Umwelt). This stability cannot be understood in terms of a fixed equilibrium, it must be considered dynamic: The system remains in constant flux, reacting to irritations from its environment. To this end, the system maintains a self-reference as its operations differentiate between what is itself and what is its environment. In social systems the process of self-stabilization is mediated through communication. Therefore, for Luhmann society as a whole consists of communication and, in fact, consists *exclusively* of communication. This communication is successful because it utilizes symbolically generalized media that are specifically useful in this situation as they secure the success of communication. Examples for such media are money, scientific truth, or power, among others (Baraldi, 2021). Within the social system sub-systems can emerge through internal functional differentiation. These sub-systems although each operationally closed, interact by irritating one another which can induce their structural coupling.^1^ The current ongoing and proposed adoption of DTs could change communication in contemporary societies substantially and is therefore of great sociological significance. Luhmann’s systems theory is a fitting model theory for this process because it can examine DTs as connecting links between system and environment in new formations. In the following we will theorize that the “structural drift” which Luhmann connects to digitalization (Luhmann, 2002, pp. 24) is ‘communicated’ by DTs as they maximize the speed of communication in a process we term systemic acceleration.

To constructivists such as Luhmann, the blueprint of a potential physical is not as accessible as that of engineered systems, and neither is their environment as sterile as in early DT adoptions. Yet, digital twins have already reached humans and the social domain, albeit not as like for like replicas. Initially, DTs merely touched the social domain tangentially in the form of the user outside their intended system boundary. Then, the concept of the DT was adopted by construction where it possibly first enclosed the social into its system boundary as users *in* rather than *of* the system (Boje et al., 2020). Subsequently, DTs were employed in urban planning where the social domain is weaved into the system much more than it is encountered in the form of the user experience in traditional products (Ravid and Aharon-Gutman, 2023). Lately, multiple projects have set out to develop DTs of the earth to combat climate change (Barros, 2024; Li et al., 2023). The foundations of such DTs are often meteorological models that are proposed to be coupled with various earth system scientific models (Li et al., 2023). Here, the boundary between society and DTs seems to finally be transcended, as some argue the coupled DTs themselves form a society (Tomko and Winter, 2019). In fact, following these arguments DTs may call for a novel notion of digital processes that are more akin to our current understanding of individual organisms (Tomko and Winter, 2019; Teller, 2021).

We think that such advances, proposing deeply intertwined systems of systems aimed at shaping future policy making need to undergo careful sociological dissemination. The rich cybernetic and systems scientific language employed by the scientific literature on digital twins motivates us to look at DTs from a Luhmannian perspective and ask the following research questions that are guided by Luhmann’s theory:

1. How can Digital Twins be characterized through a system theoretic Luhmannian perspective?
  - (Umwelt) Where in the social system are digital twins situated?
  - (System) How can the processes of digital twins be viewed through social systems theory?
  - (Kommunikation) How can the interaction of DTs and the social be characterized?
2. How would such a novel perspective shape the future of digital twins?
3. How does a Luhmannian perspective on digital twins shape our vision of a digitized society (5.0)?

This article is intended to provide a theoretical basis for further study, which can only be outlined here. In the following sections we will first construct a frame of reference that gives us the theoretical basis for applying this concept to specific case studies. To this aim, Luhmann’s central ideas and concepts are briefly outlined for this context and discussed in the section 2 for a theoretical sociological analysis of digital transformation, specifically in the case of DTs. We argue sociologically that digital twins should not be defined merely as improved computational models, but as an intensified form of systemic self-observation based on accelerated feedback loops between digital models and social systems. We discuss the effects of DTs on society on of our views on DTs and their development in section 3. Finally, a discussion section puts our theory into a broader context and outlines pathways for future research.

## 2 A system theoretical frame of reference for DTs

Luhmann’s understanding of social systems is fundamentally based on communication (Luhmann, 2018). Because to Luhmann social systems consist exclusively of communication, the central task of sociology is precisely to examine the various forms of communication in societies. More specifically: Sociology, according to Luhmann, is not an independent discipline, but rather an interdisciplinary project, the outcome of which will be a theory of society (‘Theorie der Gesellschaft’). This does not refer to a specific society, such as Germany, but always relates to global society (‘Weltgesellschaft’). Conceptually this also does not refer to agents, but to communication: “Only communication can communicate” (orig.: “Nur die Kommunikation kann kommunizieren“) (Luhmann, 2021a, pp. 105). This type of ‘Gesellschaftstheorie’ aims at a society without a specific definition of society in the narrow sense; rather, it concerns communicative processes through which society is always constituted only temporarily. Technological innovations such as DTs should be regarded as new forms of communication.

Let us take a brief step back to the basic line of Luhmann’s thinking and focus on the social transition from pre-modern to modern societies, especially the identification of characteristic modes of communication. These are autopoietic forms in the sense that they are compatible with other modes of communication, they emerge out of themselves and re-produce themselves; the difference lies in multiplying and increasing the self-referential forms. Modern societies are characterized by symbolically generalized media, that allow this autopoiesis and take shape in functionally differentiated societies: “In this transition, the symbolically generalized media worked as catalysts in the formation of some functional systems (the political system, the economic system, the scientific system, the system of families, the art system), in which the success of communication depended on the distinction between acceptance and rejection” (Baraldi, 2021). It remains to be clarified how digital communication relates to symbolically generalized media and whether this gives rise to a new society, a problem which Luhmann thinks about in *Die Gesellschaft der Gesellschaft* (Luhmann, 2021a, b). As we recall, Luhmann replaces the cartesian and kantian subject by self-referential modes of communication. Therefore, to identify the situation of DTs in the social system we focus on its self-reference, because these are the operations by which systems observe themselves. Accordingly, it is the development of functional sub-areas, with their respective codes, that makes modern societies so special. There is no center in this society, there are instead different arenas in which society is constructed (‘Polykontexturalität‘). This polycontexturality shapes the requirements the sub-areas adhere to a social DT. For example, the scientific system values accuracy and reproducibility most. In contrast, in the economic system speed can mean a competitive advantage even at the cost of some accuracy. Yet again, the political system requires a coherent narrative and political legitimacy.

Across these sub-areas, a DT shares with its physical twin a system boundary that defines the DT’s functional realm. The DT receives information about the state of the PT and its observable environment. In turn, it can influence the PT’s communication. Thus, from a systems theory perspective, DTs expand communication in several areas of society simultaneously. This means that a clear categorization based on binary codes is no longer possible. For example, in science truth/lie, in economics profit/loss, or in politics power/powerless. Instead, the DT could become an object of inter-systemic structural coupling, that simultaneously (co-)irritates multiple functional sub-systems. This raises the question of whether familiar sub-areas are still adequate for describing the complexity of contemporary society.

### 2.1 Digital twins and computer models of social systems

Through the internet and smart devices, computer models are slowly moving into all areas of social interaction and modify also their relationships. Digital models are in the political system, economics, science, transport, sport, art and so on. A Luhmannian perspective on DTs is instructive because it draws attention to new self-observations in current forms of communication. Precisely because in these cases communication constructs DTs’ forms from within through the DTs distinguishing themselves from others, DTs are an impressive link for understanding the trajectory of self-referential social systems in the digital age.

Luhmann had already addressed digitalization in his last major book *Die Gesellschaft der Gesellschaft* in the context of a theory of society: Digital communication will transform social life in much the same way as the invention of writing did in ancient times and as the invention of the printing press did in modern times (Baecker, 2006). Consequently, Luhmann imagines a completely new society that will emerge from the shift to digital communication. This transformation emphasizes the distinction and impacts of a computer society in contrast to printing press society; “[Luhmann] looks at society at large having to deal with unprecedented amounts of data stored, and processed, and computed upon with an equally unprecedented speed, both the amount of data and the speed of its computing having to be handled by communication in stock markets, in hospitals, in engineering, in natural sciences, and on desks at home and in offices” (Baecker, 2007, pp. 411).

Computer models of socially relevant systems are where we connect digital twins to Luhmann. We understand them as a product of systemic self-observation. They are, by origin, coupled to the social system and cannot but feed back into policies that affect it. The cybernetic foundation on which these models stand is deep. In fact, there is a term that predates that of the Digital Twin that directly refers to cybernetics: Cyber-Physical Systems (CPS).

**Figure 1:**
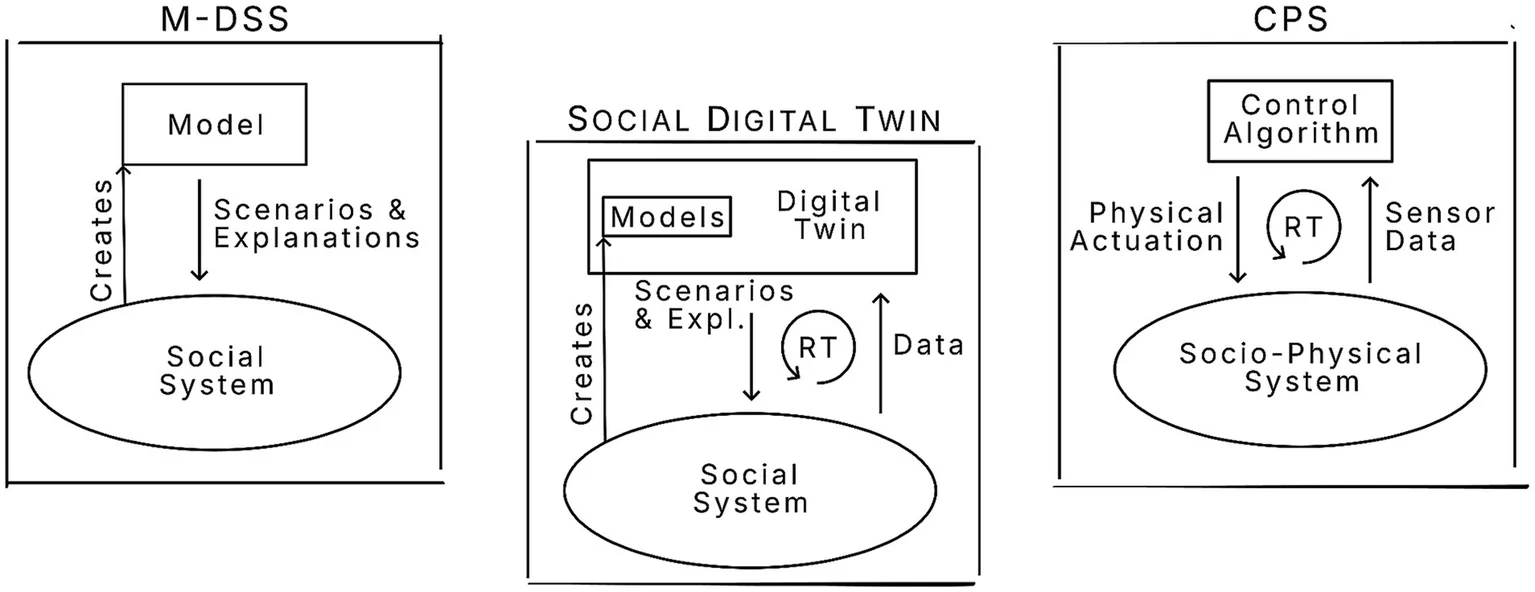
The social digital twin in the context of model-based decision support systems (M-DSS) and cyber-physical-systems (CPS). The DT adopts the real-time (RT) nature from the CPS but acts toward the social system like a M-DSS and is an integral component of the social system.

The difference between digital twins and cyber-physical systems can be inferred from the emphasis their names give to their subject. Both operate on the interface between a physical and a digital part. Digital twins focus on the digital part of the physical-digital relationship. In contrast, CPS focus on its physical interactive components such as robots, self-driving cars or the things in the internet of things. The feedback loop that defines DTs, can also be observed in CPS. However, while an intervention in the scope of DTs is an information flow back from the digital to the physical twin, in the case of CPS this interaction takes place physically. Moreover, the DT includes a detailed model of the system and its environment and considers scenarios using this model in its planning stage, whereas CPS follow a much simpler control scheme. In this sense, the difference between CPS and DTs is akin to that between first- and second-order cybernetics (see also Figure 1). Some even place DTs as an addition to CPS (Eckhart and Ekelhart, 2019). The CPS, to them, entails the digital (cyber) component as well as the physical component of the system. In contrast, the DT describes purely a digital component, despite its definition depending on the existence of a physical twin. The DT can then support the CPS and become an integral part of it (Eckhart and Ekelhart, 2019). Still, such a DT must maintain its defining capabilities that go beyond what was traditionally understood as CPS.

The revelation that the social system has to be taken into account when discussing CPS has led to the notion of Cyber-Physical Social Systems (CPSS) and Cyber-Human Social Systems (CHSS) (Kato et al., 2023). A theory of a duality of fast administrative and slow political operations in the social systems is the basis of CHSS. The CHSS exercises normative control in the fast and interacts supportively in the slow dynamic (Kato et al., 2023). It may, however, accelerate both.

The digital twin acts as the physical’s modeled identity. In the context of engineered systems, they gain the ability of self-observation. This is the smartness of smart things. The economic benefits of such thinking things are manifold. Errors must no longer unfold in the realm of the physical but are shifted to the virtual world. Risks, as products of probability and damage, tend toward zero as the physical damage becomes digital, repairable within milliseconds by pressing an undo button. What is more, the virtual world obeys a different economy of movement. Thanks to multiprocessing, contrary to its physical twin, a digital robot arm can move in multiple directions simultaneously. Various scenarios of different futures can be probed, and lessons can be learned from their simulated consequences. In an ideal, noiseless environment, all this information can then flow into a single, perfectly orchestrated and economic move on the physical side.

If digital twins add self-observation to things, what purpose do they serve for already self-observing systems (i.e., Luhmann’s social systems)? For individuals, one prominent proposal is that of a medical DT of their bodily functions (Popa et al., 2021). It would be the logical successor to the currently common regular medical check-ups. Instead of regular haemograms as individual data points in time they could relate to other medical information and, most importantly, a dynamical model of the patient’s bodily functions. Such a digital twin serves as an extension to an individual’s self-observative capabilities. It increases their context awareness and data resolution. For a sick individual it could tell the likely causes of their demise. But would the digital twin itself feel sick? Some visions of DTs seem to suggest so.

In this spirit, what is the body mass index (BMI) and all the data about average human bodies but an overly primitive precursor to a digital twin of obesity? We feed it the information about our body, with modern smart scales at a rate that can exceed daily updates, and in return we receive recommendations. Fitness apps build on this model; they add sleep scores and fitness ages and recommend workout routines and such. They are thus, in some sense, digital twins. They act as personal trainers, just as DTs could act as personal assistants that relieve us from making daily decisions (de Kerckhove, 2021). Similarly, we have had digital models of societal concern for a long time. These were commonly referred to as Model-based Decision Support Systems (M-DSS). However, in contrast to DTs M-DSS were usually designed once and then only occasionally updated while in use if at all.

The most recent example of large scale M-DSS is the management of the Covid-19 pandemic, which also saw first proposals of using digital twins for pandemic management (Stenseth et al., 2023). But other examples go back much further. Models of global population growth (Lutz et al., 2001), or the economic system (Frankel, 1986) come to mind. By our working definition, these predecessors of digital twins are, thus, an integral part of our modern society ever since we started using computers to inform our policies, lacking only the automated immediacy that we will show to be integral to real DTs. Within our framework DTs distinguish themselves from traditional model-based decision support systems in precisely these aspects: The amount of data utilized and their operational speed. The defining feature of DTs is their apparent real-time and high detail representation of their respective physical twins using emerging big data resources, the enormous amounts of data harvested via the internet. Thus, we think that DTs are, already to Luhmann, the logical consequence of the interaction between the digital and self-referential systems. They are likely to invade large parts of the social system and have the potential to majorly transform its structure by accelerating their capabilities of self-observation.

This can be understood as an approach to answer research question 1. a: Where in the social system are digital twins situated? DTs are the epitome of the accelerated social self-reference, a systemic acceleration driven by the digital revolution. They can be understood as a collision of M-DSS and CPS (see Figure 1): In terms of M-DSS they adopt the real-time feedback loop and turn the model into a digital mirror of society. From the CPS point of view, a model (or more than one) is introduced and used to evolve their first-order cybernetic control-scheme into a true means of self-observation. Thus, DTs are not external to the social system but integral components of its structure.

### 2.2 Digital twins in the social system

DTs are dependent on their physical counterpart. Thus, they cannot be considered an independent system but must instead be viewed as an outcome of systemic stabilization. Their structure is a biproduct of the autopoiesis of the PT and its acceleration, a mimicry of self-organization, mirroring self-organization with the other as the self. But as the digitally twinned physical systems interact, their interaction becomes part of a DT-to-DT coupling. This interaction is driven by its own necessity; in Luhmann’s terminology a structural drift. To follow the development of their physical counterparts as closely as possible, the DTs need to anticipate their perturbations. This information can be estimated by DTs of other systems and integrated as additional data streams. The DTs become their own system (of systems) that needs to undergo constant reconstruction to maintain its legitimacy within society. Their objective is to remain useful. Their usefulness is dependent on the accuracy of their predictions and their relevance to policies of interest. To prove the latter, they need to have an observable positive impact on decision making. However, a strong impact necessarily leads to a boundary where the impact of the DTs affects their own predictions. At this point, the DTs require self-reference to maintain the required accuracy. Consequently, a DT can never fully grasp the complexity of the model system as it aims to approach the physical twin in a never-ending convergence. This interdependency will be further explored in the next section.

DTs are plagued by a deep conflict between their proposed and their actual capabilities. In contrast to the highly controlled environments and the artificial nature of their original realm in engineering, social systems and their environments are noisy and chaotic. Their future is unforeseeable and any attempt to forecast their state is cursed to fail. Nevertheless, as described above, digital models are and have been successfully utilized in decision making in many different social systems. Their usefulness seems to withstand being challenged. Despite not painting an exact picture of the future, they can still reveal important information about *possible* trajectories and cause-effect relationships. What is more, Bayesian methods can propagate uncertainties from data into their results. This is a crucial feature of any predictive system. The quantification of uncertainty lets us distinguish between what we know and what we do not (yet) know. It can point to knowledge gaps and exposes the very complexity of the system one is trying to model. For example, an epidemic DT faces high uncertainty as a novel pathogen arises, At first, the biology may be unknown and information about the epidemiology may be sparse. The DT then needs to expose this uncertainty and its sources and point to possible clinical research that can eventually feed back into the DT’s knowledge about the pathogen and reduce the initial uncertainty.

Despite being a twin of the social system, the model employed in the DT cannot be a direct replica of the social system. Rather, it is a purpose made simplified projection. Regardless of its simplifications the systems trust (‘Systemvertrauen’) in DTs seems to be implicitly strong in common visions of DTs. This may be out of necessity: All their data originate from the very system that employs them and the accuracy of the DTs’ predictions is their very argument of existence. We will see that, despite Luhmann’s concerns of its demise (Luhmann, 2021a, pp. 312), expert knowledge is still necessary in these computer systems.

Uncertainty is not only linked to data but affects all observation. Thus, model selection, the choice how to make use of the data, must also be subject to scrutiny. Models differ in the assumptions they make. That is, a model can encode assumptions about how observations can be explained, but also about which details can be neglected in favor of computational cost. Due to the complexity of the social system, these assumptions are necessary and necessarily political. A model encodes a narrative. That is, it represents an abstracted, simplified representation adjusted to fit to selected observations of interest. Each abstraction trades possibly valuable insights for simplicity. Such apparently neglectable details can have catastrophic effects when they are disregarded. For example, during the Covid-19 pandemic model based decision support systems repeatedly failed to predict the eventual epidemic impact (Ioannidis et al., 2022). Among other reasons, this was caused by poor data and modeling assumptions and exposed both by mechanistic and by data-driven models (Ioannidis et al., 2022). Thus, the choice to include a behavioral response in an epidemiological model and how to define its parameter range is normative.

Mechanistic models encode domain specific knowledge. In this sense they are expert mediated systems such as Newton’s or Maxwell’s equations, biased by the observer’s partial albeit knowledgeable valuation of the constitutive mechanisms. For example, Maxwell’s equations describe the wave nature of massless particles but ignore their particle properties like single-photon emission. In their application to social systems (Ahamer, 2012) they naturally cannot account for individual disobedience. Similarly, the Ising model, a microscopic model of ferromagnetism, models the spin of its atomic constituents as two distinct states ignoring other spin possibilities (Ising, 1925). The same mechanistic idea is used to model voting behavior and societal opinion dynamics (Sznajd-Weron and Sznajd, 2000; Baldassarri et al., 2023). In a Luhmannian scope this model can be understood to encode communication on a network of agents who irritate one another along its links and eventually converge to a stable opinion. Naturally, without an extension to more states, it thereby ignores any other option in voting dynamics. Similarly, traditional epidemiological models did not include an asymptomatic infection that was a defining feature of the Covid-19 pandemic. These examples highlight the normative power that lies within a mechanistic model in DTs.

Mechanistic models use data to trim their parameters to the observed dynamics. This process is classically viewed as an optimization problem: The *correct* parameters are those that make the expert defined mechanics most closely resemble the data empirically. In contrast, data-driven models are entirely powered by big data and do not depend on detailed mechanistic expert knowledge. Their intrinsic behaviorism comes at the cost of the model becoming a black box. As Luhmann envisioned (Luhmann, 2021a, pp. 312–13), the trust in experts is replaced by trust in the system. This inhibits the observation of the DT’s processes reducing second to mere first-order observation and contradicting the very argument for society to create a DT. Yet, the data selection and collection processes are still largely domain driven and continue to encode biases. Data shaping the models leave their biased imprint in the model behavior and lead to, due to systems trust (‘Systemvertrauen’) seemingly objective, biased truths (Ntoutsi et al., 2020). Data-driven models can integrate new data on the fly, learn and adapt to changes in the observed system. Similarly, a DT can update the parameters of a mechanistic model in real-time. Consequently, two metrics of DT quality arise: Prediction speed and prediction accuracy. These qualities are constrained by a respective trade-off. As models become more accurate, their computational demands increase. However, given the above found acceleration imperative governing DTs, it is possible accuracy will eventually be traded for speed. Computers have long been much faster at processing information than individual humans, but they seem to lack in anticipative detail. As it stands, once computer models would be scaled up to DTs as envisioned by Grieves and Vickers (2017), encoding atom level resolution, they would lack processing power to keep up with the physical at all. The lower bounds of their accuracy are governed by their existence being dependent on the social systems that employ them and their systems trust. For example, a DT using a data-driven model that inaccurately predicts an epidemic is useless. It becomes slightly more useful if it is accurate: It can then act as a response tool where one can react to its prediction; but only if it goes beyond its black box nature and offers a good explanation for its prediction can it really feed back into society and inform sustainable mitigation and management strategies.

Novel methods, such as physics-informed machine learning, allow a combination of mechanistic and data driven models (Meng et al., 2025). In fact, a DT can combine data-driven and mechanistic models and their predictions in an ensemble of models (Gregory et al., 2024; Langlotz et al., 2022), as often used for forecasting task (Wu and Levinson, 2021). This mode of operations moves the DT into the position of an active meta-model that mediates between other models as model management system (Gregory et al., 2024). In addition to integrating data from other DTs and, thus, other models or model ensembles, the DT then compresses the knowledge creation process between possibly conflicting world views encoded in models into one narrative. This role can be delegated to a subsequent data-driven model (Langlotz et al., 2022). This externalizes the selection process that is usually negotiated within the social system to the DT, effectively giving the DT or its author full control over its narrative and, by extension, its physical twin.

As DTs of different social (sub-)systems start to interact with one another, they need to integrate the traded data in meaningful ways. In mechanistic models they need descriptive mechanisms of how the model reacts, thus the communication among DTs can be limited. Data-driven models can integrate estimates of perturbations through learning from observations. Consequently, a self-anticipating system of DTs emerges and merges into one integrated DT of society.

So, to answer research question 1. b (How can the processes of digital twins be viewed through social systems theory?) we have to remain critical about the difference between the proposed and the actual capabilities of DTs of social systems. If DTs are seen as digital mirrors, we must keep in mind that mirrors can distort images and what one sees in a mirror is what one is looking for and at. A DT can create and reproduce biases and has normative power. Nevertheless, the processes of DTs, that is, the models that link the inflow of information to its feedback into society, remain a portal for said society’s self-reference. To maintain their relevance, DTs must strive for accuracy and precision in their models, but, from the scope of social systems science, the ultimate pinnacle of a perfect model, a one-to-one relationship between a physical and a virtual reality cannot be reached. We think that the constituents of the DTs structure and operations, models and data, encode power relationships. To prevent DTs from limiting the selection space of social systems the model and data space need to remain open, interpretable and user dependent. We will explore the relationship between physical and digital twins more closely in the next section.

### 2.3 A highly accelerated structural coupling

There is a well-defined boundary that differentiates the digital twin as our system in focus from the social system that it mirrors and which simultaneously forms its systemic environment. The interaction of both systems, an integral part of the DT’s definition, is what transcends this boundary. Luhmann points us to the critical realization that social systems cannot be steered, but only irritated (Luhmann, 2021b, pp. 789). Hence, the engineering-derived idea that the feedback loop from the DT to the PT is closed via a specific and direct *control loop* loses operationality in the social domain. Instead, it must be adapted for the DT’s use in the social sphere. The DT can feed information through its sensors from the social system into its knowledge base. However, the opposite direction of the feedback loop is more intricate. In some instances, the actuation can be immediate. For example, an urban DT can predict an arising traffic jam and mitigate it through intelligent signaling. In other if not most cases, human mediated policy-steps stand between the DT and the PT. These are often referred to as human-in-the-loop. In the common DT-concept MAPE (Monitor, Analyze, Plan, and Execute) the last stage is then replaces by Suggest (Walton et al., 2026). In terms of social systems theory this means that the DT informs the social system in its feedback loop which irritates it such that its resonance is to design policies. In this process the social system remains in control of the digital twin and its operation as it self-observes through the DT. Therefore, the social DT is not a digital twin in the strict engineering sense. In the social DT, the bidirectional feedback loop between the DT and the PT refers to a highly accelerated structural coupling rather than a direct cybernetic wire.

Even the above example of an urban DT controlling traffic lights has humans-in-the-loop: A red traffic light potentially governed by a DT can be ignored by an ambulance driver in an emergency. This exemplifies how much more complex the social sphere is, even if we disregard disobedient or spontaneous behavior. However, such a DT could just as well be integrated with a health care management DT and steer both ambulance, traffic lights, and hospital staff, such that a hospital with sufficient capacities is reached as quickly as possible and already prepared for the incoming patient.

**Figure 2:**
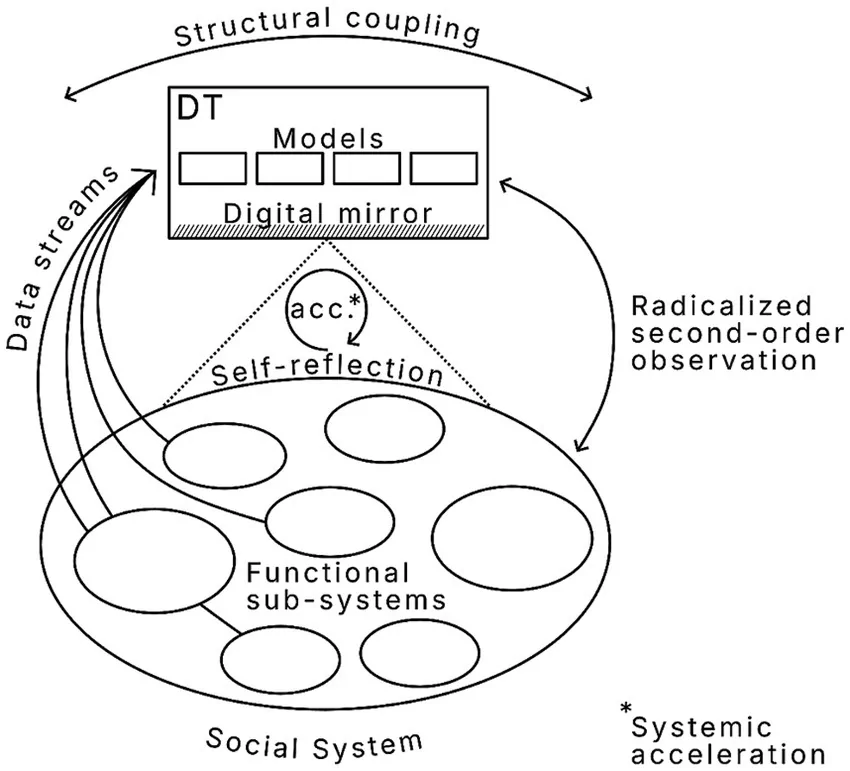
Interaction between the digital twin and social sub-systems and its effects. The real-time nature of the DT drives systemic acceleration to a point where self-reference is immediate. It starts acting as a digital mirror which radicalizes the second-order observation of the social system and facilitates structural coupling among sub-systems.

Thus, we think the interaction of DTs and the social can be characterized (see RQ 1. c) not only through an acceleration of the M-DSS feedback loop or the *smartification* of CPS. As shown in Figure 2, systemic acceleration creates a tight bond between the DTs and the social system where they act as a digital mirror. Through this structural integration arises the radicalization of systemic structural coupling that passes through the DT and is (nearly) devoid of any delay as is its social system in its self-observation. In effect, the DTs weave themselves into the social system by means of their integral part in the structural coupling. Hence, the interaction of the DT and the social system cannot be understood in terms of original engineering-derived definitions but must adhere to this function of radicalized second-order observation.

## 3 Application and implications

Having outlined our social systems-science-based understanding of digital twins, their processes and their situation in the social system it is logical to ask for the implications of our theory. We will proceed by looking into the effects of the accelerated self-reference that is driven by the DTs. Then we will discuss the future of DTs and of the society 5.0 that will embed them.

### 3.1 Infinite regress against the Lucas critique

The DT’s narrative and irritative effect should not be subject to centralized control, because that would break down its communicative effect entirely by reducing second to mere first-order observation. Second-order observation arises from the predictive nature and combinatory logic of the digital twin. Between the information we feed it and its results stands a model or even an ensemble of models. Through these means the social system is able not only to observe itself but also to observe its self-observation. Critically for a DT, this second-order observation happens in real-time, which stimulates self-reflection and, thereby, establishes a boundary with the surrounding environment. A peculiar situation of radicalized second-order observation through a digital mirror without (almost) any delay emerges. This radicalization creates a feedback loop of irritation that stands in the way of the DT’s promise: The complexity of such a situation makes it impossible for the system to fully understand itself. This problem is reminiscent of what is known in economics as the Lucas critique (Lucas, 1976): The observation that any policy derived from a model immediately renders that model invalid. In other words, because the social system reacts to being modeled the model by its own virtue invalidates itself. The self-reflection of the social system means that this invalidation can never be anticipated through the DT.

Lucas’ critique is what the constitutional acceleration in the concept of the digital twin attempts to counter. The DT tries to outrun the model’s invalidation by integrating a real-time interdependency between itself and the PT. Through this tight coupling with the modeled system, policy effects are immediately integrated into the model. In effect, the DT can be understood as a reaction to the Lucas critique, although it can never fully eliminate it. As mentioned above, the physical limitations of information flow impose a constant information asymmetry between the physical and the digital twin. As an effect of the social systems temporalization, both are doomed to run after one another in an infinite regress of second-order observation (von Foerster von Foerster, 2003) that cannot be resolved but by accepting that the Lucas critique remains valid.

For example, an epidemiological DT could (correctly) predict the onset of an influenza wave and, thereby, motivate individuals to take precautions. In effect, the influenza incidence would decline, and the DT would no longer predict the wave, which increases incidence as less caution is taken, which makes the DT predict a wave and so on in infinite regress. The DT’s predictions appear inaccurate and the problem that arises for systems trust is obvious but could possibly be resolved through oscillation damping. As another example, an epidemiological DT could predict a decline of cases after a voluntary intervention and give susceptible individuals a false sense of security. Some may decide to ignore the intervention and, thereby, trigger a rise in cases. In turn, this would lead to stricter interventions which could discourage compliant behavior to which the DT may react with even stricter policies. Here, the possibly escalatory nature of a feedback loop in infinite regress becomes evident. Of course, a DT could anticipate such effects, but what would be its solution? It would need to predict a less positive result of the intervention, which would again affect the policy effects and drive it down another path of infinite regress. What we want to show with this example is, that the DT can never be exact, no matter how fast it reacts to its own effects on the complex social system.

There is a certain danger that the DT’s infinite regress will develop into an arms race against time itself. The DT internalizes the Lucas critique and accelerates the structural evolution of society. This constant pressure on the social system to adapt its structure may accelerate the structural drift to its terminal velocity, where no further acceleration is possible. The velocity of the DT to PT interaction is limited by the processing speed of meaning processing systems. Once this is anticipated, the arms race moves to the information transmission speed in the feedback loop. As the velocity of information is physically limited by light speed, the objective must become the minimization of physical distances. Effects of this arms race are already visible: In one of the early adopters of technologies akin to societal DTs, the trade market, this momentum led to server farms moving consecutively closer to the stock exchange to maintain a competitive advantage (Frino et al., 2014). Thus, as the distance between the digital and the physical twin eventually converges toward zero, the systems become one. This raises concerns about a connection imperative arising out of the integration of digital tools in modern societies (see also Karppi et al., 2021). Such a trend was already visible in the solutionism of the Covid-19 pandemic, where digital contact tracing apps were presented as a tool for public health (Maschewski and Nosthoff, 2022). As such a tool can only work if sufficient individuals are using it, concerns of mandated app use are warranted.

We argue that DTs can never fully accurately predict the future and must maintain the same for our own predictions. However, we think that there is a strong indication that the future of DTs, as an answer to research question 2, is one of a highly integrated system that acts as a possibly distributed (physical) yet central (systemically) gateway for systemic structural coupling. Our prospect is, that this integration is self-reinforcing and ties both systems closer together as time progresses. Even though their models can never fully capture the social system, future DTs will have to constantly strive for increased accuracy in their predictions. Ultimately, the future of DTs will be determined by their own usefulness to social autopoiesis.

This view has direct implications for the design of a social DT. According to our Luhmannian analysis one can start by constructing either a M-DSS or a CPS. In the first case, the crucial change to become a DT is to adopt a real-time data stream and an adaptive model or even model management system. In the second case, the real-time information flow is already present but the predictive and analytical capabilities of a highly capable model are absent and need to be introduced (see also Figure 1). In contrast to the original target systems of DTs, the social system is not engineered but autopoietic. Thus, in DTs it is also vital to allow for a selection process that is mediated from within the original social system. In other words, the human(−s)-in-the-loop must remain in control of the DT’s narrative. As stressed above, only if the engineering derived direct cybernetic wire is substituted by such a pathway for second-order observation can the DT operate with and within the social system (see also Figure 2).

### 3.2 Accelerated self-interaction and its implications

The outstanding feature and novelty of the social DT is the acceleration and quality of its feedback loop. This acceleration is especially noticeable in the history of this feedback loop. The central role of the feedback loop in DTs points to their origins in engineering and even deeper roots in cybernetics (Wiener, 1950). In fact, it was the original theory of cybernetics that popularized the notion of a feedback loop for systemic control (Nosthoff, 2026, pp. 58). This narrative, in a similar fashion stemming from an engineering mindset, but at first applied to psychological systems, was soon extended to the control of social systems. Already these earliest applications of feedback loops on governance emphasized the real-time character and followed an imperative of acceleration to increase their theorized control efficiency (see Nosthoff, 2026 for example p. 183, 194, 222, 310ff).

The digital twin’s roots in cybernetics offer a strong potential for critique (Nosthoff, 2026). Technology is often presented as a politically neutral means that should be judged by the ends it is used for. However, the control narrative carried within cybernetics, and by extension DTs, frames their application as an optimization problem rather than an input to a discursive process. Thus, technology itself must be constructed to support the structural fabric of its constitutive social system. In other words, democratic societies must ensure their democratic control over their DTs.

Our working definition of DTs, despite the feedback loop narrative being reminiscent of a centralized control stream, does not preclude the application of DTs in democratic societies. In fact, there is no need for the feedback loop to close in a tight fashion of directly advising a select group of policy makers. This flow can just as well be distributed, heterogeneous and user dependent. Neither is it necessary for the digital twin to depend on a sole model, specific data and bias. As alluded to above, a DT can be understood as a meta-model, a model management tool (Gregory et al., 2024), that brings together models and data and reveals their synthesized, contextualized and/or counterfactual information to its users. For example, an epidemiological DT could show different predictions of the epidemic progress in context of the underlying assumptions. Users can then decide which model to trust based on their individual experience and knowledge about society and adapt their behavior accordingly.

The anticipated acceleration of its integration in the physical, social system, calls for a maximization of the information flow. Following Luhmann’s understanding of information, the system must meet the data stream with a selection of meaning. Thus, the information flow is limited by the speed of this meaning selection process. Hence, possibly contrary to intuition, wide public accessibility of data does not increase this flow but rather demands it as it increases the quantity of data putting pressure on the social system’s selection of meaning. Instead, the flow can only be increased in both directions, effectively hypersensitizing the physical system to its self-irritation via the digital. In other words, Big Data, the maximization of the flow volume out of the system cannot accelerate the information flow without an increase of the systemic inflow volume even if the data are preprocessed and compressed by the DT. In a centralized instance this is a hard limit determined by the limits of the central entity’s selection of meaning. However, in a society, the selection process can be distributed. If users can interact with the digital twin individually and control its data and model selection criteria the information flow grows substantially. Thus, the maximization of information flow between DT and PT can be the result of the DT’s democratization. However, the opposite is not necessarily true: Systemic acceleration, the maximization of communication’s speed, does not necessarily entail democratization. In the example of the epidemiological DT, despite distributed access it would not be democratic if users had no free choice to select and trust models and data.

The emerging decentralized resonance can disrupt sub-areas of the social system. If the DT’s models and biases are not contextualized among their alternatives and its processes remain opaque, nonreflective self-affirmative selection can lead to polarization. It could radicalize the political system by driving its natural fractured narrative and disrupt the scientific system that relies on a standardization. Many of the abovementioned processes are already observable in the effects of social media. Information (including misinformation) flows much faster into the system through these distributed networks than via traditional, more centralized media. The heavily unregulated nature of the new media drives self-affirmative selection and polarizes society. Using our example epidemiological DT, a large fraction of society may rightfully trust a vaccine to be an effective countermeasure to a spreading pathogen. A small fraction of society may trust another model and appear to validate it based on the vaccination of the other fraction and its effect on herd immunity. These effects should be anticipated when designing DTs of democratic systems.

We think that, due to their normative power DTs need to be carefully designed when used by democratic societies. A well-managed democratic access to DTs can foster their imperative systemic acceleration, but the integration of DTs in a society does not necessarily support democratic institutions. Thus, DTs of democratic social systems will likely have to go hand in hand with a democratic discourse on their design, which again highlights the radicalization of second-order observation that social DTs entail. We cannot pretend to predict in detail the effects of social DTs on a future of a society 5.0. However, from a systems science perspective we can respond to RQ 3 by arguing that the functional sub-systems are likely to experience stronger structural coupling. In effect, DTs have a major potential to change the structure of social systems. In the process toward a new digitized society outlined by Luhmann (2021a, b) and highlighted by Baecker (2006) we argue that DTs are the final push toward a radicalization of the effects of digitalization.

## 4 Discussion

To reiterate, in our understanding of Luhmann’s social systems (Luhmann, 2018) any computational model that is utilized, be it by scientists or decision markers, is intrinsically embedded in the overarching system and, thus, by popular definition a DT. Yet, to us the conceptualization of DTs goes beyond re-baptizing the well-established M-DSS. It captures an acceleration that is driven by the progress of digital transformation. In other words, the emphasis on *dynamical updates* and *bidirectional interaction* in their definition by the National Academies of Sciences, Engineering, and Medicine (2024) points to the potential that lies within DTs: Their power to accelerate decision support to a velocity that can match the rapid evolution of digital societies. A process that we term systemic acceleration. In fact, Luhman himself argued that the storage, processing and accessibility of data on a global level accelerates the communication to a velocity that would be impossible without computers (Luhmann, 2021a, pp. 412). If the invention of writing and the printing press perturbed the social system to evolve toward its second-order observation (Baecker, 2006), DTs accelerate this self-observation to its terminal velocity. In other words, they are the final push to a radicalized fully optimized self-observation of which further acceleration is impossible.

The theorized systemic acceleration affects all digitally modeled systems in a feedback relationship with physical systems. Thus, digital twins are bound to eventually invade all aspects of the social system. This invasive breadth of social DT technology works in unison with its overarching acceleration imperative. As meaningful information flow becomes the bottleneck in further speed increases it can only be increased by hypersensitizing the socio-technological interface. This sensitivity is directly proportionate to the *bidirectional* information flow between both entities. In the physical-digital direction the flow of sensor data into the DT is already vast. In the other direction the so-called *control flow,* that tends to spark visions of DTs as centralized authoritarian government machines, can be optimized by resisting this vision and following the opposing momentum of decentralized resonance. However, such a momentum may also lead to self-reaffirming feedback loops of individuals akin to contemporary social media echo chambers. To counter this effect, the DT must offer contextualization of its models and data and maximum transparency in a user mediated meaning selection process.

We think that DTs must be widely accessible and the power relationship must be tuned toward their users through control over both the data and the model selection of the DTs. However, the acceleration imperative puts all involved systems under pressure to process information more efficiently. Anticipative care must be taken regarding the trade-off between speed and accuracy, which can be understood as a direct consequence of the conflict between the binary codes of the economic and scientific sub-system. A DT must, therefore, expose what it does not know to the user, for example using Bayesian inference methods to offer a transparent insight into biases and uncertainties. The speed limit that bounds this acceleration is the inevitable self-reference of the digital twin. Nothing can overtake itself for it would surpass light speed as the terminal velocity of information. However, the same limits exist for the acceleration of societal evolution. What is more, this theoretical limit assumes absolute efficiency of the participating meaning processing systems. We identify their meaning processing speed as the limiting factor. This leaves the distance between the DT and its PT as the last resort for optimization, leading to its eventual diminishing. Simultaneously, DTs interact among themselves to improve their accuracy effectively integrating into one large, coupled DT of society. Ultimately, we expect one integrated hybrid socio-technological system to emerge, where the balance between an imperative to connect and a participative system of distributed interaction may be difficult to maintain.

The core differentiator between a mere computational model embedded in the social system and a DT is the emphasis of the feedback loop between the physical and the digital and its acceleration. Using live sensors and a dynamic and modular approach to modeling could give rise to a fundamental change in supported decision making. Hence, substantial transformative power lies within the adoption of societal DTs. Proposed DTs of society are envisioned to help decision makers find pathways toward prosperity and societal development (Chircu et al., 2023). Digital Twins of social equity could help to shape a future with equity as a key metric (Chircu et al., 2023). There is, however, also an imperative to take caution. Ethical considerations point to great risks that come with such a transformative technology (Fontes et al., 2024). DTs will indivertibly be biased, be it in physical models through methodological bias or in data driven models with biased data (Fontes et al., 2024; Rasheed et al., 2020). Similar inequalities already have considerable effects on society today. When DTs accelerate the digital transformation further they may increase the forces driving the digital divide (Fontes et al., 2024).

The discussion of the ethical implications of this technological innovation should be viewed from a functional angle in terms of systems theory. Moral action cannot, by its very nature, be defined in substantive terms, but is rather another example of Symbolically Generalized Media in modern society. From a systems theory perspective, DTs must be considered new forms of communication that hold a specific position in a digital society. The development of DTs and their integration in the social fabric both must happen under the consideration of systems theory. From this perspective, the DT can be understood as a tool to enhance the social system’s self-observation. It enables the physical system to observe itself and its observation process in real-time. However, its inner workings, even if they are theoretically tractable as in Stenseth et al. (2023), obscure the response of the DT. Neither can the physical system anticipate the digital’s messages, nor can the digital twin foresee what data it is to receive. To retain second-order observation, transparency is vital. Popular data-driven black box models break this expectation of the DT, forcing the social system to blindly trust it. The DT then reverts from a digital mirror of society to an opaque oracle, a modern imagination of Delphi. Full systems trust (‘Systemvertrauen’) in such a DT would replace communicative critique and pose a serious threat to a democratic society.

Furthermore, the change in reflexivity that emerges from this acceleration is of great significance in current societies. With the increasing use of DTs in social life, both interpersonal relationships and the societal forms of self-reflection are changing - aspects that will require further sociological research in the future. It is precisely from this perspective that an updated connection to the Science & Technology Studies (STS) could be achieved; however, with a focus on the close relationship between technology, society, politics, and science, practical applications of reflexivity would then have to become center attention. In this context, reflexivity could become a crucial element of a future sociology that also seeks to include AI in its field of view - dealing with DT could be the defining factor here. The key point here is that, from Luhmann’s perspective, we are considering a theory of society that needs to discuss digitalization within the context of its frame of reference. This is different from highlighting exemplary practices in a society that increasingly defines itself through digitalization, which should be subject to future research.

In accordance with Luhmann, we do not consider computer systems, algorithmically processing information, despite occasionally being black boxes, to be meaning processing systems. Therefore, they cannot constitute a social system by themselves as this would require double contingency (Luhmann, 2018, pp. 154). We abstain from describing a system of DTs as a society as suggested by others (Tomko and Winter, 2019). Even though large language models simulate human behavior very well, to Luhmann they remain statistical models that do not process meaning. In any case, we do not think that considering DTs capable of forming a society would qualitatively affect our main conclusion: The social system employs digital models to accelerate its autopoiesis to its terminal velocity. This highlights the ongoing relevance of social systems theory in the sociology of digital transformation.

## Data Availability

The original contributions presented in the study are included in the article/supplementary material, further inquiries can be directed to the corresponding author.

## Footnotes

1 Introducing the entirety of Luhmann’s social theory would go beyond the limits of this manuscript. Instead, we will give short introductions to the core concepts used here. The reader is referred to Luhmann (2018) and Luhmann (2021a) for details. For English readers we suggest Baraldi (2021) as an introduction to Luhmann’s theory.
